# *SOX10* Mutation of Waardenburg Syndrome With Hypogonadism: A Report of 2 Cases

**DOI:** 10.1210/jcemcr/luaf339

**Published:** 2026-02-11

**Authors:** Yuying Yang, Wei Zhang, Sichang Zheng, Yiran Jiang, Lei Ye, Shouyue Sun

**Affiliations:** Department of Endocrine and Metabolic Diseases, Ruijin Hospital, Shanghai Jiao Tong University School of Medicine, Shanghai Institute of Endocrine and Metabolic Diseases, and Shanghai Clinical Center for Endocrine and Metabolic Diseases, Shanghai 200025, China; Key Laboratory for Endocrine and Metabolic Diseases of the National Health Commission of the PR China, Shanghai National Clinical Research Center for Metabolic Diseases, Shanghai National Center for Translational Medicine, Ruijin Hospital, Shanghai Jiao Tong University School of Medicine, Shanghai 200025, China; Department of Endocrine and Metabolic Diseases, Ruijin Hospital, Shanghai Jiao Tong University School of Medicine, Shanghai Institute of Endocrine and Metabolic Diseases, and Shanghai Clinical Center for Endocrine and Metabolic Diseases, Shanghai 200025, China; Department of Endocrinology, Xi’an No. 3 Hospital, The Affiliated Hospital of Northwest University, Xi’an 710038, China; Department of Endocrine and Metabolic Diseases, Ruijin Hospital, Shanghai Jiao Tong University School of Medicine, Shanghai Institute of Endocrine and Metabolic Diseases, and Shanghai Clinical Center for Endocrine and Metabolic Diseases, Shanghai 200025, China; Key Laboratory for Endocrine and Metabolic Diseases of the National Health Commission of the PR China, Shanghai National Clinical Research Center for Metabolic Diseases, Shanghai National Center for Translational Medicine, Ruijin Hospital, Shanghai Jiao Tong University School of Medicine, Shanghai 200025, China; Department of Endocrine and Metabolic Diseases, Ruijin Hospital, Shanghai Jiao Tong University School of Medicine, Shanghai Institute of Endocrine and Metabolic Diseases, and Shanghai Clinical Center for Endocrine and Metabolic Diseases, Shanghai 200025, China; Key Laboratory for Endocrine and Metabolic Diseases of the National Health Commission of the PR China, Shanghai National Clinical Research Center for Metabolic Diseases, Shanghai National Center for Translational Medicine, Ruijin Hospital, Shanghai Jiao Tong University School of Medicine, Shanghai 200025, China; Department of Endocrine and Metabolic Diseases, Ruijin Hospital, Shanghai Jiao Tong University School of Medicine, Shanghai Institute of Endocrine and Metabolic Diseases, and Shanghai Clinical Center for Endocrine and Metabolic Diseases, Shanghai 200025, China; Key Laboratory for Endocrine and Metabolic Diseases of the National Health Commission of the PR China, Shanghai National Clinical Research Center for Metabolic Diseases, Shanghai National Center for Translational Medicine, Ruijin Hospital, Shanghai Jiao Tong University School of Medicine, Shanghai 200025, China; Department of Endocrine and Metabolic Diseases, Ruijin Hospital, Shanghai Jiao Tong University School of Medicine, Shanghai Institute of Endocrine and Metabolic Diseases, and Shanghai Clinical Center for Endocrine and Metabolic Diseases, Shanghai 200025, China; Key Laboratory for Endocrine and Metabolic Diseases of the National Health Commission of the PR China, Shanghai National Clinical Research Center for Metabolic Diseases, Shanghai National Center for Translational Medicine, Ruijin Hospital, Shanghai Jiao Tong University School of Medicine, Shanghai 200025, China

**Keywords:** waardenburg syndrome, *SOX10*, gonadal function, adrenal cortex function

## Abstract

Waardenburg syndrome (WS) is a complex genetic disorder primarily characterized by auditory and pigmentary abnormalities, resulting from neural crest cell migration disorders. We reported 2 genetically confirmed *SOX10*-mutant WS cases illustrating critical management principles. Both patients underwent early auditory intervention (case 1: cochlear implantation at age 3; case 2: hearing aids from age 2), resulting in preserved age-appropriate language acquisition. Each case manifested delayed puberty with biochemical evidence of hypogonadotropic hypogonadism, necessitating gonadotropin therapy to potentiate virilization and preserve fertility. Multidisciplinary care and emerging therapeutic approaches offer hope for better management. Further studies are warranted to improve the diagnosis, treatment, and quality of life of patients with WS.

## Introduction

Waardenburg syndrome (WS) was first described in 1951 by Dutch ophthalmologist Petrus Johannes Waardenburg [1]. Since then, significant progress has been made in understanding this syndrome. WS is a disorder of neural crest cell migration, characterized by auditory and pigmentary abnormalities [2]. Early research focused on clinical descriptions and classification of the different subtypes based on phenotypic features. Over time, with the advancement of genetic technologies, the identification of disease-causing genes has become a major focus. For example, the discovery of mutations in genes like *PAX3*, *MITF*, and *SOX10* has provided insights into the genetic basis of WS. The use of techniques such as whole-exome sequencing and targeted sequencing has enabled the identification of novel mutations and a better understanding of the allelic and genetic heterogeneity of the syndrome [2].

WS is estimated to account for approximately 2% to 5% of all patients with congenital hearing loss [3]. A screening program in Colombia between 2002 and 2005 of 1763 institutionalized deaf individuals identified 95 affected individuals from 95 families, giving a frequency of 5.38% of WS among the institutionalized deaf population [4].

Intrafamilial phenotypic variability and nonpenetrance are common features in WS. For example, in families diagnosed with WS1, WS2, and WS4 with pathogenic variants in *PAX3, MITF*, and *EDNRB,* respectively, such phenomena were observed [2]. Gonosomal mosaicism for a variant in *PAX3* was seen in an asymptomatic father of 2 affected siblings, further complicating the inheritance patterns [2]. The coexistence of different subtypes of WS in the same individual, along with multiple genetic diagnoses, has also been reported, highlighting the genetic complexity of this syndrome [5].

The clinical manifestations of WS are diverse and can vary widely among individuals. Sensorineural hearing loss is a common feature, present in approximately 71.0% of patients, and is predominantly bilateral [6]. Pigmentary abnormalities are also characteristic, including white forelock, skin hypopigmentation, heterochromia iris, and hypopigmentation of the iris and choroid [4, 7]. There is an overlap in clinical manifestation and genetic predisposition between WS and idiopathic hypogonadotropic hypogonadism (IHH). Three percent of IHH patients carry the *SOX10* mutation [8]; 62% of them had at least 1 known WS-associated feature. Given the overlap in clinical manifestations and genetic background between WS and IHH, WS should be considered as a key differential diagnosis for patients with IHH, especially those with concurrent auditory or pigmentary abnormalities. These manifestations suggest that WS can affect multiple organ systems, emphasizing the need for comprehensive management.

## Case Presentation

### Case 1

**Figure 1.**
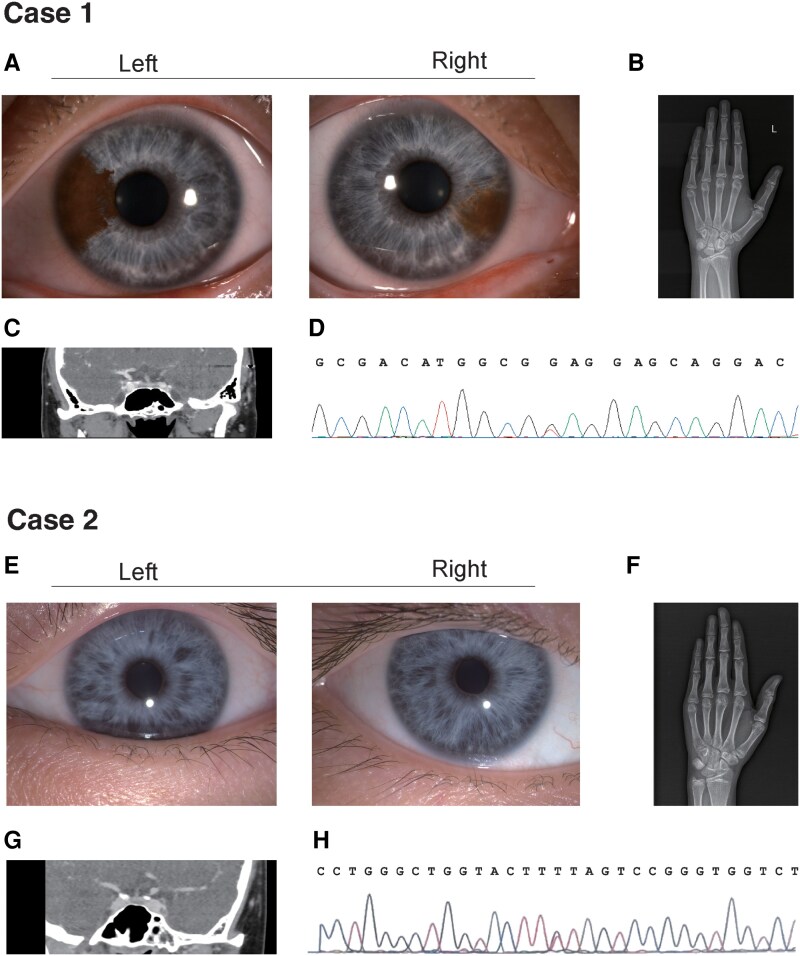
(A) Bilateral blue iris (a typical pigmentary abnormality of WS). (B) Left-hand radiograph reveals bone age of 15 years (chronological age 18 years). (C) Pituitary CT shows no obvious structural abnormalities. (D) The *SOX10* heterozygous mutation c.7G > T (p.Glu3Ter) at the seventh nucleotide position of the coding region. (E) Persistent bilateral blue iris (a pigmentary feature of WS). (F) Left-hand radiograph shows bone age of 16 years (chronological age 19 years), indicating delayed bone development. (G) Pituitary CT shows hypodense lesion in the pituitary gland with a diameter of approximately 4 mm. (H) The *SOX10* heterozygous mutation c.513C > A (p.Tyr171*) at the 513th nucleotide position. Abbreviations: CT, computed tomography; WS, Waardenburg syndrome.

Case 1 was an 18-year-old male who was born in Anhui, China, and presented to our clinic with a history of congenital deafness and delayed puberty. He was the firstborn child of nonconsanguineous parents, with a younger brother who showed no signs of WS. The patient's birth and early developmental history were unremarkable, except for the presence of congenital deafness, for which he received a cochlear implant at the age of 3. Pubertal development was delayed, with no signs of secondary sexual characteristics at age 14. At the age of 18, he presented with gynecomastia and was found to have color blindness during a routine health checkup. On physical examination, he was noted to have blue iris, no axillary or pubic hair, absence of an Adam's apple, and gynecomastia. Bone age was 15 years (chronological age 18 years), as shown in Fig. 1B. Ultrasound examinations revealed bilateral gynecomastia (mammary gland tissue thickness: 14 mm on the right side, 16 mm on the left side) and relatively small testes compared to the normal volume for an 18-year-old male (left: 33 × 21 × 15 mm, 7.38 mL; right: 31 × 18 × 13 mm, 5.15 mL) and seminal vesicle and prostate glands, with no significant abnormalities in the epididymis, spermatic cord, or bladder. Hormonal assays showed low levels of LH, FSH, and testosterone, consistent with hypogonadotropic hypogonadism (Table 1). LH-releasing hormone (LHRH) stimulation testing revealed normal LH responses (1.11 mIU/mL at baseline and 12.98 mIU/mL after stimulation; reference for baseline 0.57-12.07 mIU/mL) and FSH responses (2.07 mIU/mL at baseline and 6.04 mIU/mL after stimulation; reference for baseline 0.9-12.0 mIU/mL) (Table 2). A nonprimed insulin tolerance test (ITT) was performed, which showed low peak GH (peak GH < 3.0 µg/L) and a potential adrenal insufficiency, as evidenced by a peak serum cortisol level of 14.42 µg/dL (SI: 398.1 nmol/L) (Table 2). However, the patient had no history of fatigue, hypotension, or hyponatremia. Basal ACTH level was 36.2 pg/mL (SI: 8.0 pmol/L, reference:7.0-65.0 pg/mL, [SI:1.54-14.30 pmol/L]), and an ACTH stimulation test was not performed. Genetic testing revealed a heterozygous mutation in the *SOX10* gene: c.7G > T (p.Glu3Ter), which was confirmed as pathogenic by the ClinVar database.

**Table 1.** Basal hormone levels

	Case 1	Case 2	Reference range
ACTH	36.2 pg/mL (SI: 7.97 pmol/L)	32.9 pg/mL (SI: 7.24 pmol/L)	7.0-65.0 pg/mL (SI: 1.54-14.31 pmol/L)
Cortisol	7.32 µg/dL (SI: 202.05 nmol/L)	7.95 µg/dL (SI: 219.32 nmol/L)	6.7-22.6 µg/dL (SI: 184.84-623.49 nmol/L)
UFC	24.55 µg/24 hours urine	69.30 µg/24 hours urine	21-111 µg/24 hours urine
Aldosterone	13.662 ng/dL (SI: 37.90 pmol/L)	2.982 ng/dL (SI: 8.27 pmol/L)	2.906-33.278 ng/dL (SI: 7.96-92.31 pmol/L)
Renin	50.17 pg/mL (SI: 2.91 mIU/L)	29.96 pg/mL (SI: 1.74 mIU/L)	4-38 pg/mL (SI: 0.23-2.21 mIU/L)
GH	0.163 ng/mL	0.033 ng/mL	0.003-0.971 ng/mL
IGF-1	188 ng/mL (SI: 24.63 nmol/L)	148 ng/mL (SI: 19.39 nmol/L)	43-220 ng/mL (SI: 5.63-28.82 nmol/L)
LH	0.53 mIU/mL	<0.07 mIU/mL	0.57-12.07 mIU/mL
FSH	1.55 mIU/mL	0.14 mIU/mL	0.9-12.0 mIU/mL
Testosterone	0.26 ng/mL (SI: 0.89 nmol/L)	3.76 ng/mL (SI: 13.04 nmol/L)	1.42-9.23 ng/mL (SI: 4.92-32.04 nmol/L)
SHBG	17.83 nmol/L	40.87 nmol/L	13.5-71.4 nmol/L
T3	2.08 nmol/L	1.96 nmol/L	0.54-2.96 nmol/L
T4	130.01 nmol/L	109.75 nmol/L	62.67-150.84 nmol/L
FT3	4.92 pmol/L	5.99 pmol/L	2.43-6.01 pmol/L
FT4	13.49 pmol/L	13.33 pmol/L	9.01-19.04 pmol/L
TSH	0.9567 mIU/L	1.4237 mIU/L	0.3500-4.9400 mIU/L

Abbreviations: FT3, free T3; FT4, free T4; UFC, urinary free cortisol.

**Table 2.** Provocative test results

GnRH stimulation test							
	−15 min	0 min	25 min	45 min	90 min	180 min	
Case 1							
LH	1.11 mIU/mL	1.25 mIU/mL	12.03 mIU/mL	15.06 mIU/mL	12.98 mIU/mL	8.55 mIU/mL	
FSH	2.07 mIU/mL	2.04 mIU/mL	3.88 mIU/mL	5.40 mIU/mL	6.04 mIU/mL	5.94 mIU/mL	
Case 2							
LH	/	<0.07 mIU/mL	0.84 mIU/mL	0.81 mIU/mL	0.58 mIU/mL	/	
FSH	/	0.37 mIU/mL	2.18 mIU/mL	2.75 mIU/mL	2.54 mIU/mL	/	
HCG stimulation test (case 1)							
	−15 min	0 min	24 hours	48 hours	72 hours		
Testosterone	0.46 ng/mL (SI: 1.59 nmol/L)	0.44 ng/mL (SI: 1.53 nmol/L)	2.27 ng/mL (SI: 7.87 nmol/L)	4.25 ng/mL (SI: 14.74 nmol/L)	5.69 ng/mL (SI: 19.73 nmol/L)		
Insulin tolerance test							
	−30 min	0 min	30 min	45 min	60 min	90 min	120 min
Case 1							
ACTH	61.45 pg/mL (SI: 13.53 pmol/L)	25.86 pg/mL (SI: 5.70 pmol/L)	14.37 pg/mL (SI: 3.20 pmol/L)	17.27 pg/mL (SI: 3.81 pmol/L)	204.18 pg/mL (SI: 46.00 pmol/L)	10.17 pg/mL (SI: 2.24 pmol/L)	16.95 pg/mL (SI: 3.73 pmol/L)
Cortisol	15.01 µg/dL (SI: 414.09 nmol/L)	9.46 µg/dL (SI: 260.98 nmol/L)	6.98 µg/dL (SI: 192.56 nmol/L)	6.34 µg/dL (SI: 174.91 nmol/L)	12.30 µg/dL (SI: 339.33 nmol/L)	14.42 µg/dL (SI: 398.02 nmol/L)	9.55 µg/dL (SI: 263.47 nmol/L)
GH	0.62 ng/mL	0.129 ng/mL	0.125 ng/mL	0.166 ng/mL	0.803 ng/mL	0.436 ng/mL	0.142 ng/mL
Case 2							
ACTH	34.11 pg/mL (SI: 7.51 pmol/L)	24.01 pg/mL (SI: 5.29 pmol/L)	71.44 pg/mL (SI: 15.78 pmol/L)	295.41 pg/mL (SI: 66.05 pmol/L)	199.05 pg/mL (SI: 45.06 pmol/L)	30.97 pg/mL (SI: 6.82 pmol/L)	24.08 pg/mL (SI: 5.31 pmol/L)
Cortisol	7.91 µg/dL (SI: 218.22 nmol/L)	6.18 µg/dL (SI: 170.50 nmol/L)	6.55 µg/dL (SI: 180.70 nmol/L)	20.75 µg/dL (SI: 572.45 nmol/L)	20.73 µg/dL (SI: 571.90 nmol/L)	14.57 µg/dL (SI: 401.96 nmol/L)	11.82 µg/dL (SI: 326.10 nmol/L)
GH	0.121 ng/mL	0.435 ng/mL	2.891 ng/mL	2.328 ng/mL	1.427 ng/mL	0.294 ng/mL	0.077 ng/mL

Abbreviations: HCG, human chorionic gonadotropin.

### Case 2

Case 2 was a 19-year-old male who presented to our hospital with a history of growth and pubertal development delay. He was born at full term via normal delivery to nonconsanguineous parents, with blue iris noted at birth but normal vision. His early developmental milestones were notable for speech delay and hearing loss diagnosed at age 2, for which he received hearing aids. The patient has a healthy 15-year-old younger brother with normal development. At age 13, the patient was noted to have absent secondary sexual characteristics with underdeveloped testes and penis. Physical examination revealed persistent blue iris, small testes as determined by ultrasound (left: 11 × 6 × 9 mm, 0.42 mL; right: 12 × 6 × 11 mm, 0.56 mL), and delayed pubertal development. Karyotype analysis confirmed 46,XY male genotype. Initial hormonal evaluation showed markedly low testosterone (<0.08 ng/mL) (<0.28 nmol/L, reference: 1.42-9.23 ng/mL, [SI: 4.92-32.04 nmol/L]) (Table 1). GH during ITT demonstrated suboptimal responses, with a peak GH of 2.328 ng/mL (Table 2). LHRH stimulation testing revealed blunted LH responses (<0.07-0.84 mIU/mL) and low FSH responses (0.37-2.75 mIU/mL) (Table 2), consistent with hypogonadotropic hypogonadism. Treatment with human chorionic gonadotropin (HCG) (1000 IU twice weekly) and GH therapy was initiated in November 2019, resulting in a 20 cm height increase (from 140 to 160 cm) over 3 years. His height reached the genetic potential (father: 162 cm; mother: 150 cm; predicted: 162.5 ± 5 cm). Genetic evaluation at age 14 identified a heterozygous stop-gain mutation in the *SOX10* gene (c.513C > A:p.Y171*). Recent evaluations showed persistent hypogonadism (LH <0.07 mIU/mL, FSH 0.14 mIU/mL, testosterone 3.76 ng/mL [SI: 13.04 nmol/L]) after treatment. Testicular ultrasound demonstrated minimal growth (left: 16 × 7.0 × 14 mm, 1.11 mL; right: 15 × 8 × 15 mm, 1.28 mL) with a right epididymal head cyst. ITT showed low peak GH (peak <3.0 µg/L) with preserved adrenal function (cortisol >18 µg/L [SI: 496.4 nmol/L]). Pituitary computed tomography showed hypodense lesion in the pituitary gland with a diameter of approximately 4 mm. Bone age was 16 years (chronological age 19 years), as shown in Fig. 1F.

## Diagnostic Assessment

### Case 1

Clinical manifestations were congenital deafness and bilateral blue iris. Basal hormonal evaluation indicated hypogonadotropic hypogonadism (Table 1, Table 2). ITT showed low peak GH (peak GH <3.0 µg/L) and subnormal cortisol responses. Sanger sequencing identified a heterozygous mutation in *SOX10*: c.7G > T (p.Glu3Ter) (Pathogenic, ClinVar accession: VCV000995928.3).

### Case 2

Clinical manifestations were congenital deafness and bilateral blue iris. Hypogonadotropic hypogonadism was confirmed by low basal testosterone and blunted LHRH responses (Table 1, Table 2). ITT showed low peak GH (peak GH <3.0 µg/L). Whole-exome sequencing identified a heterozygous *SOX10* mutation: c.513C > A (p.Tyr171*) (pathogenic, ClinVar accession: VCV003601843.1).

## Treatment

Case 1 initiated gonadotropin therapy (HCG 1000 IU) twice weekly via intramuscular injection along with calcium and vitamin D supplementation. Case 2 continued gonadotropin therapy (HCG 2000 IU twice weekly intramuscular injection) along with calcium and vitamin D supplementation.

## Outcome and Follow-up

Case 1 did not come back for follow-up. Case 2 came back to our hospital 7 months later. Testicular volume (measured by ultrasound) increased from 1.11 mL to 1.23 mL (left) and 1.28 mL to 1.30 mL (right). Testosterone was 3.09 ng/mL (SI: 10.72 nmol/L) which was slightly lower than before (3.76 ng/mL [SI: 13.04 nmol/L]).

## Discussion

Currently, there is no cure for WS. However, supportive techniques can alleviate some of the symptoms. Cochlear implantation is a common approach for patients with profound bilateral sensorineural hearing loss, which is a major symptom in WS [3]. A meta-analysis of cochlear implantation in WS patients showed that there are no significant differences in the scores for categories of audit performance, speech intelligibility rating, and parents' evaluation of aural/oral performance of children between WS patients and non-WS patients, indicating its comparable efficacy [9].

Multidisciplinary care models are essential for the management of WS patients. Given the diverse clinical manifestations and potential multisystem involvement, a team approach can provide comprehensive care. Similar to other complex genetic disorders like Moebius syndrome and Prader-Willi syndrome, a multidisciplinary team for WS could include audiologists, ophthalmologists, geneticists, speech and language therapists, and psychologists [10, 11].

Monitoring and managing gonadal function are equally important. Gonadal dysfunction is common in certain hereditary endocrine disorders, which may manifest as underdeveloped gonads or abnormal sex hormone secretion [12]. For male patients, it is necessary to assess testicular function, including testosterone levels and fertility. For female patients, attention should be paid to the regularity of menstrual cycles and changes in estrogen levels.

Here we document 2 genetically confirmed *SOX10-*mutant WS cases illustrating critical management principles. Both patients underwent early auditory intervention (case 1: cochlear implantation at age 3; case 2: hearing aids from age 2), resulting in preserved age-appropriate language acquisition. Each case manifested delayed puberty with biochemical evidence of hypogonadotropic hypogonadism, necessitating gonadotropin therapy to potentiate virilization and preserve fertility. Case 1 showed subnormal peak cortisol (14.42 µg/dL [SI: 398.1 nmol/L]) during ITT but lacked clinical symptoms of adrenal insufficiency and confirmatory data from ACTH stimulation tests. Thus, this finding should be interpreted cautiously and requires further validation. GH deficiency is not a known feature of WS. Peak GH <3.0 µg/L may not be sufficient to diagnose GH deficiency, and further studies are needed to explore potential associations.

Our 2 cases further confirm the association between *SOX10* mutations and hypogonadotropic hypogonadism, which aligns with previous studies [8]. *SOX10* is a transcription factor critical for neural crest cell development and migration—cell populations that contribute to the formation of the hypothalamus and pituitary gland, key components of the hypothalamic-pituitary-gonadal axis. Pathogenic *SOX10* mutations lead to reduced secretion of GnRH from the hypothalamus or gonadotropins (LH, FSH) from the pituitary. This mechanism explains why both patients presented with delayed puberty and low basal LH and FSH, as well as the need for HCG therapy to promote virilization. Notably, 3% of patients with IHH carry *SOX10* mutations, and 62% of these individuals have at least 1 WS-related feature [8]—reinforcing that WS should be a key differential diagnosis for IHH, especially in patients with concurrent auditory or pigmentary abnormalities. Notably, for case 1, GnRH stimulation showed normal LH/FSH responses, which do not exclude constitutional delof puberty (CDGP)—a key overlap, as CDGP patients often retain intact pituitary responsiveness to GnRH. However, critical factors support hypogonadotropic hypogonadism: the patient presented at 18 years with persistent delayed puberty (no secondary sexual characteristics by 14, low testosterone), highly atypical for CDGP (usually resolves by 14-15 years). Additionally, his pathogenic *SOX10* mutation provides a genetic basis for hypothalamic-pituitary-gonadal axis dysfunction.

These findings expand the WS phenotype to include neuroendocrine dysfunction and mandate endocrine surveillance beyond gonadal evaluation, particularly given the potential for life-threatening adrenal crisis during physiologic stress.

## Learning Points

- Early auditory intervention is critical for preserving age-appropriate language acquisition in *SOX10*-mutant WS.
- *SOX10*-mutant WS may associated with multiaxis endocrine abnormalities, requiring comprehensive endocrine evaluation and targeted treatment.
- WS should be a key differential diagnosis for IHH, especially in IHH patients with concurrent auditory or pigmentary abnormalities.
- Multidisciplinary care is essential for the comprehensive management of WS.

## Contributors

All authors made individual contributions to authorship. Y.Y., W.Z., S.Z., and Y.J. were responsible for the diagnosis and management of this patient and manuscript preparation. L.Y. and S.S. were responsible for the analysis of genetic data and interpretation of the findings. All authors reviewed and approved the final draft.

## Data Availability

Original data generated and analyzed during this study are included in this published article.

## Abbreviations

CDGP: constitutional delay of puberty
HCG: human chorionic gonadotropin
IHH: idiopathic hypogonadotropic hypogonadism
IT: insulin tolerance test
LHRH: LH-releasing hormone
WS: Waardenburg syndrome
